# The Diseases of the Diaphragm in Man

**Published:** 1847-07

**Authors:** 


### ART. XI.

*Die Krankheiten des Zwerchfells des Menschen.* Von C. W. MEHLISS, M.D., &c.—*Eisleben,* 1845.

*The Diseases of the Diaphragm in Man.* By C. W. MEHLISS, M.D., &c.—*Eisleben,* 1845. 8vo, pp. 212.

THE diagnosis of disease of the diaphragm is attended with so much difficulty and uncertainty, that every attempt made in an earnest and philosophical spirit practically to advance our knowledge of the subject, or even to collect and arrange the scattered and fragmentary information afforded by medical literature, is deserving of praise. We ought, indeed, to consider ourselves the more beholden to Dr. Mehliss, that this task, which he has undertaken and fulfilled with great industry, and, upon the whole, with much good sense, is not altogether a very grateful one. We were really not a little curious, at first, to ascertain how it could be possible to fill 212 pages with a subject of apparently so little promise. Still, as we proceeded, our interest, we confess, soon became sufficiently excited to carry us very pleasantly to the conclusion of the treatise. It contains little, perhaps not any, information altogether new, but it has brought the scattered rays into such a focus as to render them, in some degree, available for practical purposes. It reteaches us a lesson which the progress and bent of anatomical pathology in the present day had almost made us forget; namely, that the diaphragm, far from being a mere blank leaf between two all-important classes of organs, is a muscle whose mere functional derangement may seriously, if not irreparably damage both the one and the other; in fine, that its condition ought always to be carefully investigated and duly weighed in our estimate both of thoracic and of abdominal disease.

The work commences with an elaborate account both of the anatomy and physiology of the diaphragm, in which, however, there is little that requires comment, or that is not to be found, in a condensed shape, in every good hand-book of anatomy. From this portion, therefore, we shall merely quote as useful the observation of Krause,* "that the highest convexity of the diaphragm, in the adult, is on a level with the upper margin of the middle portion of the seventh rib, or with the cartilage of the fifth; but that on the right side, it rises from half an inch to an inch higher than on the left."

* Handbuch der Menschlichen Anatomie, Vol. I. Hanover, 1838.

Since the publication of Dr. Mehliss's work—in short, quite recently—a most valuable and interesting paper has been read at the Medico-Chirurgical Society,* on the physiology of respiration, wherein it is shown by the author, Mr. Hutchinson, contrary to the generally received opinion, that, whilst ordinary, involuntary respiration is performed chiefly, if not solely, through the instrumentality of the diaphragm, this organ remains nearly quiescent during a profound respiration. Of the truth of this the reader may convince himself by experiment. He will find that every *ordinary* act of inspiration is accompanied by a relaxation of the abdominal muscles, denoted by a protrusion of the abdomen; every ordinary act of expiration by their contraction. Now it is to be remembered that, in breathing, the action of the abdominal muscles is inverse or antagonistic with that of the diaphragm. In the above movements the intercostal and other respiratory muscles participate only very slightly, so that a large proportion of the lungs does not become inflated. On the other hand, all the intercostal and other auxiliary muscles of respiration are engaged during a profound, or it might be more correct to say, a *full* inspiration, whilst the abdominal muscles are tightly braced up, the hypochondria drawn inward, and the diaphragm, if anything, slightly relaxed. Indeed, so long as the diaphragm is under contraction, it would seem to be impossible for the upper portion of the thorax to expand. We must, however, add to Mr. Hutchinson's statement, that after full expansion of the upper portion of the chest, and consequently inflation of the peripheral portions of the lungs have been effected, the diaphragm may, by a *voluntary* act, be still made to contract, by which means no inconsiderable amount of air may yet be inhaled. This double act of inspiration is, however, obviously a forced one, and not very frequently performed.

To remind the reader that both surfaces of the diaphragm have a serous investment would be needless, except, perhaps, for the purpose of remarking incidentally what is sometimes lost sight of, namely, that the base of the pericardium is firmly attached to the central tendinous portion of the diaphragm, by means both of cellular tissue and of fibrous bands, which close connexion, of course, tends very materially to promote a reciprocal reaction between the heart and diaphragm, both in health and in disease.

The position of the diaphragm, the importance and variety of its functions, the sensitive character of the momentous organs and parts by which it is compassed, or traversed, render it apparent how seriously disease of this muscle must impair the general health, but serve at the same time to explain both the obscure and the dependent nature of most of its ailments. The multiplicity of its sympathies, resulting not alone from juxtaposition, but also from the numerous excito-motory influences to which it is subject†, has gained for it a reputation for an extraordinary amount of inherent muscular irritability. No other muscle of the human body is so greatly under the influence of reflex action as this. Respiration, laughter, sobbing, coughing and sneezing, and again vomiting and defecation, are severally enacted by the whole or parts of the diaphragm sympathising

* Since published in the Medico-Chirurgical Transactions.

† The phrenic nerves are often derived from the anterior branches of the second to the sixth cervical nerves—always from the third and fourth—and are at their origin often connected with the cervical ganglia of the great sympathetic, with the pneumo-gastric nerve, and with the descending branch of the hypoglossus. Besides this, the diaphragm receives emissaries from the solar plexus.

indirectly; that is, through the medium of the nervous centres, with remote nerves directly stimulated. These are, for the most part, involuntary acts; for, although the muscle is to nearly an equal extent under the control of the will, this power is here more frequently exercised in arresting or subduing, than in promoting its action. Ordinary respiration would, however, appear to be the only function in which the diaphragm is singly active. In all its other functions it is associated with other muscles, and that not in consequence of any direct nervous consensus between them. All are here evidently set in motion directly from the sensorium, and not all simultaneously, but in the exact sequence in which they derive their nerves from the cerebro-spinal centres. Thus, in laughter, the muscles of the face receive the impulse first; and if the latter be slight, exclusively. Where the impulse is greater, the laryngeal,—then the pectoral muscles,—next the diaphragm,—and lastly, where the impulse is violent, the abdominal muscles severally, or all, become engaged.

*Disease of the diaphragm.* If we except the heart, no muscle of the human body is probably more often morbidly affected than the diaphragm. The variety of its affections is, however, not commensurate with their frequency, the great majority ranging between the extremes of spasm and paralysis. Of these two, spasm is by far the more common, the number and extent of its nervous sympathies causing the organ to participate very readily in spasm affecting other parts, whilst these identical relations at the same time prevent its entire innervation from becoming easily suspended. Thus, in general convulsions of whatever kind, the diaphragm is always in a high degree implicated, whereas, in general states of paralysis, its activity is, for the most part, little impaired. The other affections of the diaphragm are nearly all due or subordinate to, or at any rate inseparable from, the diseases of adjacent parts. Upon the whole, thoracic disease is more readily communicated to the diaphragm than abdominal; the latter has, however, a much greater tendency to excite in it convulsive action.

The symptoms generally indicative of diaphragmatic ailment are divisible into those of *anomalous sensation* and those of *anomalous motion.* The former consist in a characteristic sense of tightness or constriction at the lower part of the thorax (which is also felt sympathetically, from organic disease of the cervical portion of the spinal marrow); and, secondly, in pain, which, in partial disease of the diaphragm, is referred to the affected locality,—in general disease of the organ, to behind the inferior portion of the sternum, and from thence across to the spine. This pain is never, as some writers affirm, very intense, and differs in no particular from muscular pain elsewhere.

*Anomalous motions of the diaphragm.* These are tremulous, spasmodic, or convulsive. The preternatural sensations they produce have a singular effect upon the imaginations of patients, who often fancy that some living animal has taken up its abode in their interior;—notions which may possibly have given rise to the fables recounted from time to time of serpents being extracted from the stomachs of men. The vibratory movements of a tremulous diaphragm are not unfrequently perceptible to the eye.

*The secondary symptoms* of diseased diaphragm consist in disturbance of those functions which are carried on through its instrumentality or aid.

Here, of course, respiration is principally concerned. In slighter affections of the diaphragm, ordinary breathing may proceed without obstacle, and the difficulty be felt only when the necessity for a deeper inspiration occurs. Where the affection is more considerable, ordinary respiration becomes more abrupt. In disease of the organ attended with pain and fever, both conditions are observable, respiration being short, and at the same time frequent, hurried, the patient feeling, on an attempt to inspire deeply, as if the lung were held fast. Under these circumstances, the breathing sometimes becomes so hurried as to exceed in rapidity the arterial pulse, when it may be likened to the panting of hounds heated with the chase. Where again the diaphragm is disabled by abnormal action, respiration is abrupt, interrupted, irregular in rhythm. Where its *activity* is altogether suspended, the results depend upon the circumstance as to whether it be or be not still susceptible of passive motion. In the former case, as, for example, in paralysis of the diaphragm, the abdominal muscles in some measure act for it, and we have for a while the phenomenon of so-called abdominal respiration, the real import of which we shall afterwards endeavour to explain. In the other case, as in tetanic states of the diaphragm, for instance, complete apnœa ensues, which would necessarily terminate in death, if the other muscles of respiration failed to take the entire office upon themselves. Other cognate functions are equally liable to interruption or disturbance from the same causes; laughing, coughing, sneezing,—speech itself—being thereby restrained, or at least modified in a very peculiar manner. Finally, the power of defecation is liable to suspension, the want under these circumstances being felt, but the ability to satisfy it withheld.

*Changes in the position of the muscle, and in its relation to other organs and parts.* When under the influence of abnormal contraction, the diaphragm withdraws the cartilaginous appendix of the sternum, and the common margin of the cartilages of the false ribs, to which it is attached, towards the centre of the thoracic cavity. By this means a groove is formed externally, about the hypochondria, which is rendered somewhat deeper by inspiration, but flattens during expiration. "The heart's pulsation," says our author, "then becomes less distinct." This is, however, chiefly the case during the former act, which, under these circumstances, is accomplished by the pectoral muscles, causing the thorax to expand depthwise (instead of lengthwise), and the heart therefore to recede momentarily from the pectoral walls. The conditions are, in fact, the same as during the full natural inspiration before described. Total inaction of the abdominal muscles during respiration is a positive sign of suspended mobility of the diaphragm. It is most apparent in painful affections of the organ and, when, protracted, commonly leads to vesicular emphysema of the lungs, owing to the inadequate performance of the act of expiration.

The ancients believed in a much closer intimacy between the brain and the diaphragm (the φρένες of the Greeks) than the present standard of our knowledge of neuro-pathology suffers us to admit. It is, however, to be observed that this belief passed current up to a comparatively recent period, and we think it not quite improbable that the tricks of the earlier mesmerists may have been founded upon this traditional error. At the present day we are no longer on the watch for wild delirium to confirm our suspicions of a severe lesion or other affection of the diaphragm, nor do we, on the other hand, take the latter for granted merely because the patient's facial muscles are seen to be distorted with the so-called sardonic grin.

In the rarer instances of independent disease of the diaphragm, the symptoms before specified will probably be sufficiently marked. Where this is not the case, however, the author sagaciously employs the method "by exclusion." Thus, in doubtful cases, he is in the habit, *provided there be no obvious counter-indication,* of trying the effect of sternutatories. The patient who can sneeze vigorously cannot possibly be labouring under any severe affection of an organ whose entire freedom of action is indispensable for the performance in question. Nor is the converse less true; the total incapability to sneeze, assuredly implies suspended mobility of the diaphragm.

*Wounds and contusions of the diaphragm from external injury.* The importance of these, for the most part, merges in that of the simultaneous lesion of contiguous parts. They are inflicted by means either of firearms or of some sharp instrument, and more often through the thorax than through the abdomen. Mere contusions of the diaphragm may occasion internal hemorrhage or inflammation, and eventual gangrene of the organ. Penetrating wounds are, however, for the most part of a much graver character. Where their locality and size are such as to admit of speedy cicatrization, they are, so far as they are alone concerned, seldom productive of much mischief. In the opposite event, either they cicatrize imperfectly, and eventually give rise to diaphragmatic hernia (strictly so called), or else they remain permanently open, affording to a greater or smaller amount of abdominal viscera a free passage into the thorax. The consequences are permanent disturbance to the thoracic organs, and, sooner or later, fatal asphyxia. This prolapsus of the abdominal organs is more frequent when the left side of the diaphragm is wounded; on the right side, the liver opposes a decided obstacle to such a consummation. To this part of the subject we shall afterwards have to recur.

*Cases.* An instance is cited from Schmucker* of a hussar who received two lance wounds, one in the left inguinal region, the other, immediately afterwards, in the epigastrium, which penetrated to the depth of an inch and a half, obliquely in the direction of the diaphragm. Violent diaphragmatitis ensued, being preceded by a night passed in mingled *laughter, crying, and vomiting.* Under vigorous antiphlogistic treatment, the patient recovered, so far as to appear able to return to his duty. At this epoch, however, he was unfortunately taken prisoner, marched away on foot, and lost sight of.

In this case, the diaphragm was supposed to have been wounded but not pierced. Instances of penetrating wounds of this organ are related by several of the older medical authors, amongst others, by Morgagni. In some of these the chief symptoms recorded, namely, orthopnœa and vomiting, only terminated with death; in others, seeming recovery was followed, after a longer or shorter interval, by deadly symptoms of incarcerated bowel. An interesting case is related by Boyle, in the 'Edinb. Med. and Surg. Journ.' in 1812. A man, about 40 years of age, was,

* Wahrnehmungen.

after a meal, seized with shivering, nausea, and vomiting; the abdomen was retracted, sunken. He was largely bled, and took a dose of castor oil, which, however, the stomach rejected. The employment of a warm bath was followed by two copious stools; nevertheless the vomiting continued, and death ensued within twenty hours. The stomach was found so enlarged as to fill the entire abdomen. On its removal was seen the diaphragm, presenting a large convexity downwards, and in its fleshy portion a slit through which the jejunum, the greater portion of the ileum, and the transverse colon, together with the greater omentum, had penetrated into the left thoracic cavity. The intruding viscera were enveloped by the omentum as within a sac, and strangely intertwisted. The edges of the wound in the diaphragm were smooth and of a ligamentous character, the transposed intestines not incarcerated, but, in the neighbourhood of the wound, attached by means of fibrous threads to the mediastinum and costal pleura. Eleven months previously, the man had received a wound between the sixth and seventh ribs on the left side, but which had quickly healed. Six months later, he had suffered disturbance of the digestive organs, which, though transient, left him less fit for labour, and constantly afflicted with dyspnœa. "Morgagni mentions a case in which the wound in the fleshy part of the diaphragm was scarcely large enough to admit a man's little finger; nevertheless the greater portion of the colon had become protruded through it." Transposition of abdominal viscera is, however, not an invariable consequence, even of extensive and mortal wounds of the diaphragm. Gunshot wounds are, for the most part, attended with such grave injury to other internal organs, that the share taken by the diaphragm in producing a fatal issue can with difficulty be estimated. A somewhat interesting case of imperfect recovery from a gunshot-wound is told by Bilguer.* A Prussian grenadier had been wounded by a musket-ball, which entering on the left side, between the sixth and seventh ribs, passed out again on the right side, at two fingers' breadth from the third lumbar vertebra. There were no symptoms of the injury of any viscus, and the patient did well. From that time forward, however, he was unable to walk erect, or otherwise than with his body bent forward, and he was discharged from the army in consequence. Every attempt to assume an erect posture was attended with a painful sensation of tension far back in the umbilical region. It does not, however, appear to us very certain that the diaphragm was here in fault.

The following case is pretty generally received, in Germany, as evidence of complete recovery from an undoubted wound of the diaphragm. It is related by Dr. Grabl, of Hamburgh, in 'Fricke and Oppenheim's Zeitschrift' (1838). A cavalry officer stabbed himself with his sabre, which entered at the inner side of the left nipple, and passed out again one and a quarter inch from the vertebral column, and two inches above the os sacrum. The neighbourhood of the latter wound forthwith became strongly emphysematous. Symptoms of wounded lung alone manifested themselves, and perfect recovery followed within a month. Dr. Mehliss justly observes, that as no abdominal viscus appeared to have sustained any damage, the sabre may very possibly have passed through the thick layers of dorsal muscles into the external skin, and the diaphragm have escaped injury altogether. This, therefore, as well as other cases recorded, by Larrey

* Wahrnehmungen.

for example, of alleged recovery from penetrating wounds of the diaphragm, can hardly be received as fully established.*

*Rupture of the diaphragm.* By this term we would designate a separation of the muscular fibres, with simultaneous rupture of the serous investments. Actual transverse laceration of the muscular fibres is rare. The extent, attachments, and manifold as well as forcible uses of this organ, cause it to be more obnoxious to this lesion than any other muscle; and this, not alone through violent contusion of or falls upon the abdomen, but also through the straining of difficult parturition. Instances of rupture from excessive vomiting are scarcely to be relied upon. Contrary to Devergie, our author declares the fleshy portion to be far more liable to rupture than the tendinous, which, he affirms, suffers only when the accident occurs close to its confines.

Both authorities agree that the left side is incomparably more often affected than the right. It is highly probable that the protrusion of the abdominal viscera through the wound takes place with violence at the very moment of the rupture, and to a much greater extent than in the case of penetrating wounds, which would account for its being fraught with more peril, and more frequently productive of asphyxia. A good selection of cases is given, each illustrative of rupture from a different accidental cause. The first, is that of a man labouring under ascites, who fell forward upon his belly, and almost immediately died asphyxiated. The dropsical fluid was found to have entered and filled the thorax through a diaphragmatic rupture, an inch long. We have next an instance of rupture through contre-coup, in a youth upon whom a wall fell, burying him beneath the ruins. A compound fracture of the leg-bones occurred simultaneously. Death took place through asphyxia, the patient previously feeling as if something were tied round the heart. The stomach, colon, and omentum were found to have passed into the thorax, through a rupture four inches long and one and a half wide, and to have compressed the left lung. Mr. Wheelwright's case, cited from the 'Medico-Chirurgical Transactions,' vol. VI, furnishes an example of fatal hemorrhage, consequent upon rupture of the left side of the diaphragm, produced by falling from a coach. The extravasated blood filled the left cavity of the thorax. Finally, we have an instance of very extensive rupture, caused by the effort to prevent a cumbrous body from rolling down a steep descent. In animals of burden, and especially of draught, the accident is frequent from a cause similar to that last mentioned. One case is cited from the late Sir A. Cooper, and another from Percy, of the rupture occurring during *labour*. In the latter, the parturient, suddenly alarmed at seeing her husband fall from his chair, uttered a loud shriek, then feebly muttered a few words, gave birth to a child, and immediately afterwards expired. The phrenic rupture was on the left side, and five inches in length.

The above are all cases of longitudinal separation of the fleshy fibres; nor is the author aware of more than a single well-authenticated instance of their transverse laceration, namely, in a person who died from the effects of a fall, and in whom the front and sides of the diaphragm had become torn away from their sternal and costal attachments.

The diagnosis of phrenic rupture is exceedingly obscure. Since the

* The escape (in any quantity) of air through a recent *wound in the abdomen* affords positive evidence of the diaphragm having been pierced.

time of Celsus, authors have done little more than ring the changes upon his sentence. "Si septum transversum percussum est, præcordia sursum contrahuntur; spina dolet; spiritus rarus est; sanguis spumosus fertur." But in this there is nothing quite distinctive of the affection. It is a matter of surprise how such men as Larrey and Percy should have revived the obsolete fancy of rupture of the diaphragm being necessarily associated with distortion of the facial muscles. Still a sunken expression of the countenance will always be observable, where a portion of intestine has become actually incarcerated. Upon the whole, the practitioner has little to depend upon, except the locality of the pain (when present) and the presumptive evidence of transposition of the abdominal viscera, such as a sinking in and apparent emptiness of the abdomen; permanent dilatation of one side of the thorax, mostly the left; a preternaturally clear, or preternaturally dull sound on percussion; respiratory murmur, feeble or null,—borborygmi heard instead; heart's sounds obscure. Not every case of rupture, however, is attended with immediate displacement of the bowel; and again such displacement may be not recent, but of long standing, perhaps congenital; considerations of no little moment in medico-legal practice.

*Spasmodic action of the diaphragm.* No other muscle is so liable to become influenced by spasm as this. It is, however, far more frequently implicated in general spasmodic states than independently affected.

*Tremulous or vibratory motion of the diaphragm.* Many of our readers may have experienced this in their own persons. It is felt in the anterior portion of the diaphragm, giving rise to a sickly and anxious sensation. It is often both palpable and visible from without, in the shape of epigastric pulsation. Sometimes it alternates with painful spasmodic contraction of the rectus muscle, which, however, ceases on the individual bending the body backwards. Of a like nature is the trembling of the diaphragm in the cold stage of intermittent fever. Dr. Mehliss refers the phenomenon to irregular circulation of the blood through the diaphragmatic vessels.

*Convulsive action of the diaphragm. Singultus or hiccup* is produced by the periodical and sudden contraction and depression of the entire diaphragm, and the peculiar and abrupt sound that accompanies it, by air suddenly and forcibly entering at the mouth or nostrils to replenish the void caused by the sudden retraction of the root of the tongue. The exciting cause is, for the most part, irritation from the organs or parts subjacent to and in contact with the diaphragm, less frequently from those within the thorax. In animals, it can be called forth by irritating the cardiac orifice of the stomach.* We have not space to enumerate all the various circumstances that may give rise to this convulsive affection. It has, however, a fruitful source in reflex action, as instanced by the effect of intestinal worms, and the like in producing it. It may have its origin in mental emotions, or be transmitted by imitation from child to child; or attend the development or exercise of the sexual functions, or their suppression. It is a common and obstinate symptom in hysteria. All these instances are abundantly confirmed: Dr. Mehliss is, however, aware but of one authentic case of the irritation emanating directly from the roots of the phrenic nerves. It is that of Watmough, recorded in the 'London

* J. Müller, Handb. der Physiol., vol. i, 3d ed.

Med. Gaz.' for 1840, of singultus following concussion of the spine, and being overcome after six months' duration, by the application of a seton in the neck.

It is obvious from the foregoing that, as a symptom, singultus is little to be relied on, inasmuch as in one and the same disease, it may arise from a great variety of causes. Being difficult to imitate successfully, its presence may be regarded as distinctive of real from feigned spasm. Again its occurrence disproves any very grave affection of the diaphragm, whilst its being associated with pain demonstrates either the diaphragm itself, or a contiguous organ or part to be the actual seat of disease.

In no fewer than seven of the aphorisms of Hippocrates, is the occurrence of singultus during disease declared to be of evil omen, and the same belief long maintained its ground. The truth, however, is, that although in fever, as also in inflammatory disease of many of the organs and parts subjacent to the diaphragm, singultus denotes a grave character of the disorder, it cannot, singly, form the basis of any certain prognosis. Still Dr. Mehliss believes that in puerperal peritonitis, and in dysentery, the supervention of hiccup is generally prophetic of a fatal issue.

It is almost needless to state that the treatment of singultus must for the most part resolve itself into that of the disease upon which it depends. It is only where, from the violence or obstinacy of the symptom, life is either endangered or rendered burdensome, that direct treatment is admissible. But as no remedy has proved generally successful in such cases, we shall omit to specify all that have been tried, and simply refer to the sternutatories recommended by the father of medicine, and by which again Aristophanes is said to have been cured of obstinate hiccup, which we suppose the gods had afflicted him with as a punishment for the mischief which his farces had wrought upon the diaphragms of others. We will, however, also mention a strange and somewhat rude, but perfectly successful, method adopted by Cruveilhier in protracted cases. Having caused the patients to seat themselves with their heads bent backwards, he poured a continuous stream of water, to the extent of a gallon, into their open mouths. To augment the energy of the pharyngeal contractions, he from time to time allowed the water to enter at the nostrils, producing cough, symptoms of asphyxia, and convulsive movements in all the muscles of respiration. The well-known domestic methods of treatment such as frightening the patient, or endeavouring to astonish him either agreeably or otherwise, or again urging him to suppress by a voluntary effort the involuntary movement, are scarcely applicable except in the treatment of children.

*Convulsion,—clonic spasm of the diaphragm.* This is characterized by anormal contractions of the diaphragm, which succeed each other so rapidly that the muscle does not become perfectly relaxed during the intervals. For this reason the normal movements of the organ are greatly curtailed, and its functions considerably impeded. "A painful sense of tightness with vibration in the region of the diaphragm, suspension of respiration and of the power of utterance, together with a drawing in of the hypochondria, are the subjective signs indicative of these attacks, which attend general convulsions of almost every kind, whether epileptic, hysterical, infantile, or puerperal." Dr. Mehliss is, perhaps, right in deeming this affection to be generally referable to irritation of the roots of the phrenic nerves. But the irritation would be more often a reflected than a direct one.

*Tetanus, or tonic spasm of the diaphragm.* Prolonged contraction of the diaphragm, in consequence of preternatural irritation. The muscle forms a rigid septum between thorax and abdomen. Yet the degree of spasmodic rigidity varies; passive movement being, to a certain extent, still occasionally possible. Where it is not so, a deep inspiration is succeeded by complete apnœa, and this again by pain in the diaphragmatic region, and a progressive sense of anguish throughout the chest. In the former case the breathing is uninterrupted, but imperfect, occurring only through the instrumentality of the intercostal and auxiliary pectoral muscles. It appears as if the patients were forcibly and voluntarily holding their breath, or straining at stool. The seizure is for the most part sudden and unannounced by any premonitory symptoms. During the seizure the hypochondriac regions always appear furrowed. The patients are compelled to sit up and bend the chest forward, unable to utter a cry, and hardly able to make known in broken sentences their sensations. The face is pale at first, but becomes bloated and blueish if the attack is prolonged. The pulse is either natural, or else quick, small, and soft. Such are the main characteristics of tonic spasm of the diaphragm, a peculiar form of phrenic asthma, differing from "angina pectoris," in the absence of that agonising pain felt at the region of the heart in the latter disease, in which, on the other hand, the pitting at the hypochondria is wanting. Moreover, neuralgia of the cardiac plexus is rather a disease of advanced life, tonic spasm of the diaphragm one of youth.

The well-known transient pain occurring during violent exercise, and commonly termed "*stitch*," is justly ascribed by Dr. Mehliss to partial spasm of the diaphragm, and not to sudden gorging of the spleen with blood, or pressure from flatus, as many writers maintain. The speedy relief afforded by bending the body towards the affected side, supporting the part with the hand or a handkerchief tied round the body, and slowly inhaling air, all of which is practised instinctively by children confirm our author's assumption. That the right side is so seldom affected in this manner, is here again probably owing to the support derived from the liver.

That the crowing inspiration of infants is for the most part the work of reflex action can hardly be doubted, and it is probable that the motory nerves of the diaphragm are here almost simultaneously affected with the nerves of the muscles of the glottis. But there is little evidence in favour of Dr. Mehliss's assumption, that spasm of the diaphragm here precedes that of the glottis, much less that the latter is dependent upon the former. For this would imply the necessity that the diaphragm should become relaxed first, which is contrary to experience, the crowing character of the first returning inspirations and the heaving of the upper part of the chest during their performance proving that the diaphragm is not yet released. In this instance Dr. Mehliss reasons altogether from false data, for he attributes the crowing sounds, not to inspirations, but to air forcibly expired. It must be admitted, however, that much obscurity still prevails with reference to spasm of the glottis. It is certainly due to a variety of causes, and amongst others, perhaps, now and then, where there is great enlargement of the thymus gland, to pressure upon the phrenic nerves in their passage along the outer margins of this gland.

*Paralysis of the diaphragm.* This may result either from interrupted or impaired innervation, or else from destruction of its inherent muscular irritability. The latter kind, usually termed atony, is perhaps the most frequent, and at the same time most complete; for the diaphragm has too many sources of nervous influence readily to admit of their being all intercepted. Still, division of, or ligature applied to the phrenic nerves, in animals, is followed by sudden and fatal palsy of the diaphragm, as shown by Lower,* and more recently by the late Sir A. Cooper. Atony of the muscle differs in degree; when slight, it is manifest only where extraordinary exertion is used, as by panting in ascending stairs, &c., or in febrile states of the system when more severe it is conspicuous during ordinary breathing; in its highest degree it is denoted by respiration seeming to be almost entirely carried on through the abdominal muscles, by suffocative symptoms of various kinds, and finally by a double act of expiration. This grade occurs only as the closing scene to acute diseases, and is often incorrectly termed "paralysis of the lungs." With regard to the signification of "abdominal respiration" it must be admitted that much error prevails. In *complete* paralysis of the diaphragm, inspiration is effected through the agency of the pectoral muscles, whilst the abdominal muscles, by firmly contracting, fix the base of the thorax, the diaphragm having no longer the power to perform this office itself. Expiration is then, so far as the abdominal muscles are concerned, simply a cessation of those contractions,—a momentary relaxation. Hence the action of the abdominal muscles is here only antagonistic with that of the muscles by which respiration is, in reality, still carried on.

The duplex act of expiration is caused by the passive diaphragm, mechanically depressed by the inhaled air, relapsing by two steps, as it were, into its natural position. Thus, at least, Dr. Mehliss seeks to explain it. It seems more probable, however, that the first act is referable to collapse of the upper part of the thorax; the second to a return of the diaphragm to its place, through the propelling aid of the abdominal muscles.

Atony of the diaphragm may be produced by inordinate exertion of the muscle, such as climbing lofty mountains, by anæmia, by pulmonary disease, by parturition, or by spasm; or, mechanically, by the pressure of dropsical or other accumulations within the abdomen; or, as Stokes has shown, within the thorax. Where the abdomen is their seat, the evil consequence to the diaphragm is scarcely felt until after the removal of the offending matter, and the danger from this source of incautiously instituted paracentesis of the abdomen is well known.† Rapid delivery of a full-grown fœtus is not rarely fatal, for the same reason. The permanent relaxation of the diaphragm, from accumulations within the abdomen, is sometimes a source of angina pectoris, owing to the position which the liver is thereby made to assume relatively to the heart.

After the *cautious* removal (where possible) of that which caused the

* Phil. Trans., vol. i.

† Persons in whom there is a great accumulation of fat about the abdomen are for the same reason often much injured bp the use of drastic purgatives.

paralysis, and the employment of effectual means to prevent its return, we have little more than a dietetic course to rely upon for the further treatment. This should, however, include a bracing air, and the frequent use of the cold bath. For a length of time the bowels will probably not act without the aid of aperients,—in the selection of which, however, the practitioner should bear in mind that *tone* is the element most needed, —reduction of strength the evil most to be avoided.

*Interrupted or impaired innervation,* as a cause of paralysis of the diaphragm, has been as yet little investigated or even thought of. The subject is altogether a very obscure and difficult one, and the few cases cited by Dr. Mehliss, are either too imperfectly related, or too complicated, to admit of any conclusions being built upon them.

*Rheumatism of the diaphragm.* This, when occurring during the course of acute muscular rheumatism, is often extremely painful. It is, however, more frequent as a mild, independent affection, in males of a nervous, but more particularly in females of a hysterical habit. It is most common in wet seasons, especially towards the latter end of autumn, when its occurrence is sometimes epidemic. It infests ill-ventilated underground tenements, and especially the low, damp dwellings of the poor. It consists in a dull pain, extending from the ensiform cartilage and the inferior extremity of the sternum to the spine. A somewhat deep inspiration sensibly increases the pain, which, though never very acute, occasions much distress, as denoted principally by dejected spirits and a marked expression of anxiety depicted in the countenance. The tongue is generally clean, but of a blueish cast; the appetite not deficient, but even a moderate meal invariably occasions several hours of increased suffering. The bowels are sometimes obstinately costive; at other times tolerably regular in their action, which latter is, however, attended with great augmentation of the pain. The complaint, if not interfered with, will persist for weeks, if not months, and then gradually subside; in which case, however, there remains a great liability to its frequent recurrence. The heart's action is, for the most part, turbulent, insomuch that we have repeatedly seen cases which had been mistaken for organic disease of the heart. In the majority of these cases we have found it requisite to confine the patient for some days to bed, and to cause a succession of mustard poultices to be applied at intervals, alternately to the epigastrium and to the back. Warm diluent drinks, with occasional doses of Dover's powder, and enemata of warm water (purgatives being here decidedly injurious) have generally sufficed to effect a tolerably speedy cure. With respect to diet, it is necessary to restrict the patient, both as regards the quality and quantity of his food. But as transgression in either respect is attended with immediate punishment, the temptation to it is not great. Such is the disease in its uncomplicated form. We have sometimes observed the patient's urine to be quite clear, often to deposit lithate of ammonia, but scarcely ever to be so charged with the purpurates as Dr. Mehliss would teach us to believe. And here we may remark, (without intending any play of words) that whenever Dr. Mehliss details *cases* of his own, they give one the impression of being too highly coloured to resemble Nature very accurately. Portraits of disease will seldom admit of all the minuteness and finish of a Gerard Dow. There is, perhaps, no source of error either more fruitful or more common than working up to preconceived opinions!

*Inflammation of the diaphragm.* Little reliance can be placed on the accounts given of this affection by ancient authors. There is little doubt that it does occur now and then, in consequence both of wounds of the organ and of internal disease. In the latter case, however, it either originates or else merges in inflammation of neighbouring parts, so that its individual features are not sufficiently marked to offer a distinct portrait.

*Diaphragmatic pleurisy* has no other features to distinguish it from ordinary pleurisy, besides the locality of the pain and its violence during cough, the extreme dyspnœa, and, finally, the occasional accompaniment of singultus. Andral, however, mentions also the presence of nausea and vomiting, even in certain cases in which the stomach was found quite healthy. Sometimes a very distressing hiccup attends convalescence, —or adhesions take place between the lung and diaphragm, giving rise to permanent shortness of breath. When the right side is affected, the liver often becomes implicated, as is then manifested by icteric symptoms, &c. and the disease is often mistaken for hepatitis. Diaphragmatic is more perilous than costal pleurisy, on account of the greater embarrassment to the respiratory function. The treatment is that of ordinary pleurisy. The singultus attending some cases during convalescence, is said to have been successfully combated with the tincture of stramonium.

It is scarcely likely that the *peritoneal investment* of the diaphragm is ever generally and independently inflamed. This coating is not a continuous one, but is interrupted by folds, thrown off to several of the abdominal viscera, so that its extensive inflammation must needs involve that of one or more of the said viscera. Singultus, dyspnœa, phrenic pain, are common symptoms of the inflammation of organs subjacent to the diaphragm; if, therefore, we even admit the occasional occurrence of simple peritoneal diaphragmatitis, we are familiar with no symptoms distinctive of it. The disease is not unfrequently reflected from the pleura to the corresponding portion of the diaphragmatic peritoneum, which is then found, after death, to have undergone thickening and adhesions; the latter sometimes inclosing tuberculous deposits. In almost all these instances, the intervening muscular fibres appear perfectly healthy. Is not the singultus, before stated to occur now and then during convalescence from diaphragmatic pleurisy, probably dependent upon this implication of the peritoneal coat?

*Malformations of the diaphragm.* We shall confine our notice to such as would seem at all instrumental in engendering disease. *Narrowness of the foramina* deserves to be mentioned in the first place; that of the aortic foramen, recognized by Haller, may occasionally have some share in the production of thoracic aneurism. Of narrowness of the other foramina no authentic cases have been recorded; at the foramen quadrilaterum it is not very likely to occur, owing to the strong tendinous fibres by which that passage is protected. There are better grounds for believing dysphagia to be now and then a consequence of this malformation at the foramen œsophageum.

A more important vice of organization (where it can be deemed such) is that denominated *diaphragmatic hernia,* and which consists in the formation of a diverticulum or hernial sac, the short end of which is directed upwards, within the thorax, whilst the base opens into the abdomen. This sac is, for the most part, diminutive, but occasionally exceedingly capacious, and it always contains a smaller or greater proportion of abdominal viscera. The parietes of the sac sometimes consist of the serous investments of the diaphragm only,—the muscular fibres having merely undergone separation; at other times the walls include the muscular fibres, and the hernia is simply a partial distension of the organ along with its serous coats. In the majority of cases these herniæ have been accidentally discovered during the dissection of bodies, without any symptoms having even denoted their existence. In a few of the recorded instances, however, the enormous mass of abdominal organs, both hollow and parenchymatous, contained in the sac, cannot have failed to produce the greatest disturbance to the organs, both of circulation and of respiration. There are examples of a violent blow struck against the abdomen, sufficing to produce fatal hernia at the anterior portion of the diaphragm, situate between the sternal and costal portions, where no muscular fibres occur, and the two serous sacs therefore meet. Hernia occurring in the body of the diaphragm is probably, in most instances, dependent on congenital malformation.

*Ectopia, or the displacement of abdominal organs through an opening* in the diaphragm is discussed by Dr. Mehliss at considerable length, with a view to discriminate between the cases dependent on arrest of primary development and those resulting from accident or disease. Total absence of the diaphragm is only met with in the instance of acephalus. Four marked cases are cited of infants who did not long survive, and in whom the greater portion of the left half of the diaphragm was found wanting. In each the left thoracic cavity was found encumbered with an enormous mass of abdominal viscera. In the adult the inference is in favour of congenital malformation: 1st. Where the opening is very wide, and the edges of the wound smooth and even, presenting no marks of cicatrization. An acquired rent of the same extent must needs have proved speedily fatal. 2d. Where there is actual want of substance. Mere rupture could not occasion this. 3d. Where the opening occurs in the tendinous portion, where the fibres are least of all likely to give way.

The passage of pus and other fluids, or of acephalocysts through the diaphragm, is generally followed by collapse and cicatrization, or else by adhesions between the muscle and some contiguous organ, so that no permanent opening becomes established. It has been before stated, that an important symptom of extensive displacement of the bowel, consists in a notable sinking in of the abdomen. This is not, however, invariably perceptible; for it is remarkable that Nature appears bent upon compensating for the dislodged viscera, by causing the dilatation or enlargement of those that still remain. The liver, in particular, is then sometimes found to have attained a prodigious size. Of the descent of thoracic organs into the abdominal cavity, through an opening in the diaphragm, we have no well-authenticated record. Such an opening did not appear to exist in Vetter's interesting case of a vigorous man's heart being situate in the left hypochondrium, where it could be grasped with the hand, and its contractions and dilatations distinctly felt.

The question of how the opening originated is chiefly interesting in a medico-legal point of view; since the lesion itself, however produced, is obviously beyond the reach of art. Still the mere suspicion of its existence,—(for certainty there cannot be)—offers sufficient grounds for the immediate employment of those precautionary measures into which the treatment of this grave affection must resolve itself. Of gastrotomy, for the purpose of replacing the extruded viscera, though seriously suggested by Laennec,—there can surely be no further question at this time of day.

*Adhesions of the diaphragm* with neighbouring organs are frequently met with as a consequence both of superficial and latent inflammation, and of other destructive diseases. They of course seriously interfere with the functions of the diaphragm, more especially when occurring with the subjacent organs. Nothing, however, with certainty denotes their existence during life. Dr. Mehliss infers from a case observed by himself, that the circulation may become seriously embarrassed by these adhesions involving the foramina through which the great blood-vessels traverse the diaphragm.

*Parasitic formations in or upon the diaphragm.* The various kinds of eruption stated to have been found on the diaphragm, are all referable to an earlier period of pathology. We must restrain the temptation we feel to give our readers the benefit of a full-length portrait, drawn with medieval circumstance by Lanzoni, of a lascivious female, (salacissima quædam muliercula), who, being alarmed during the act of parturition (?) fell into a convulsive state, with alternate fits of hiccup and of laughter, which, after forty hours' duration, terminated in death. The diaphragm was found "undequaque vesiculis polyporum colore protuberans, quarum nonnullis phlebotomiâ adopertis, emanabat serosus liquor, colore aqueo, sed acidissimo odore imbutus." Dr. Mehliss regards this as a case of vesicular eruption,—Joseph Frank, with more verisimilitude, as one of hydatids. A remarkable case of hydatid cyst is recorded by Pohl, (Diss. Lipsiæ, 1747). It was twenty-six inches long and fifteen wide, and situate at the inferior surface of the diaphragm, which it had forced far into the thorax. A longitudinal incision gave escape to some 200 acephalocysts, of various sizes, from that of a fist downwards. The narrator believed the principal cyst to have formed through distension of the cellular tissue situate between the muscular fibres and the peritoneum. The subject was a female, aged 26, and far advanced in her third pregnancy. The trichinia spiralis has occasionally been met with in the muscular substance of the diaphragm.

But we have already somewhat surpassed our proper limits, and must needs conclude. Those amongst our readers who are familiar with the German language, will do well to procure Dr. Mehliss's work, which, upon the whole, cannot but be regarded as a valuable contribution to pathological literature.

